# Supplementary material for: Tissue-Specific RNA-Seq Analysis of Cotton Roots’ Response to Compound Saline-Alkali Stress and the Functional Validation of the Key Gene GhERF2
Source: Plants (Basel). 2025 Mar 1;14(5):756. doi: 10.3390/plants14050756 (PMC11901839; doi:10.3390/plants14050756)
Supplement: Supplementary file 1 [file plants-14-00756-s001.zip › Supplemanry Tables.pdf]

Table S1 Ingredients of complex salt-alkali

Ionic composition of saline alkali land		Composite salt alkali composition	
Ionic	Quality(mg/g)	chemical compound	The quantity of matter（mol/L）
Ga^{2+}	0.96	$CaCl_2$	0.0970
Cl^-	1.70	$NaHCO_3$	0.0086
Na^+	1.67	Na_2SO_4	0.1411
HCO_3^-	0.13	K_2SO_4	0.0097
SO_4^{2-}	5.02	$MgSO_4 \cdot 7H_20$	0.0583
K^+	0.19	pH	7.9
Mg^{2+}	0.35		
pH	7.9		

Table S2 The primer sequences used for qRT-PCR analyses

**Primer**	**Sequences(5'-3')**
GhUBQ7-F	GAATGTGGCGCCGGGACCTTC
GhUBQ7-R	ACTCAATCCCCACCAGCCTTCTGG
GH_A01G0012-F	TTCTGGTGATGGTGTGAGCC
GH_A01G0012-R	ATTTCCCGTTCTGCAGTCGT
GH_A01G0013-F	CGGACGAGGAACCTGTTGAT
GH_A01G0013-R	AGCGATGGTGCAACACATTT
GH_A01G0044-F	TCGAAGCGAAGTGTACGGTC
GH_A01G0044-R	ACCGGTGGCGGAATTGTATT
GH_A01G0081-F	TTCGGCTTATACTCCGTCGC
GH_A01G0081-R	TTAGTACGGTGGTGGGGGAT
GH_A01G0075-F	GTGGTTCGAGAATCGGAGGA
GH_A01G0075-R	GTTTGAGCCCTGTTGCGTTA
GH_D04G1581-F	AAGGTGGAAACGGGAAGGTC
GH_D04G1581-R	TTTTCAGGTGGAGGAGTGGC
GH_A01G0153-F	CACCTGTTAAGAAAGCAGAGTC
GH_A01G0153-R	TTCATCCGAAGAGCTGTCAC
GH_A01G0176-F	CACAACGATGAATGTGCCCG
GH_A01G0176-R	CGCTTGCTTTAGGTGCCTTC
GH_A01G0090-F	GAGCGTCGAAGGCTATGACT
GH_A01G0090-R	ATCCACTTTGTGGCAACCCA
GH_A09G1013-F	GTCTTCCAAGCAAGGTGGCA
GH_A09G1013-R	GATGCCTTTGCTCTCAGCTC
GH_D04G1535-F	TCTGTCAAGTGATCGGTGCC
GH_D04G1535-R	AGTCCCTCCGTTCCATCCAT
GH_D07G1274-F	CGCTGCTTCGACACATTACG

GH_D07G1274-R	TTTCCTCTCGGGGCATTGAC
GhERF2-F	GCCAAAGCGAGGCATTACAG
GhERF2 -R	CATAAGCCAAAGCCGCATCC

Table S3 Primer used to construct the *GhERF2* vector

GhERF2 -VIGS-F	CTCCAAGGCTTTGTTGAATTTTC
GhERF2 -VIGS-R	TAACACTTGATTGTCATGTGTACAC
GhERF2 -subcellular localization-F	ATGGAGATGTATCCGAGTAGCA
GhERF2 -subcellular localization-R	ACTGAATAACACTTGATTGTCATGTG
